# Peripartum Ηysterectomy: A Four-Year Obstetric and Anesthetic Experience in a Tertiary Referral Hospital in Greece

**DOI:** 10.7759/cureus.25062

**Published:** 2022-05-17

**Authors:** Michael Sindos, Konstantinos Kalmantis, Konstantinos Samartzis, Michail Diakosavvas, Andreas Kalampalikis, Konstantina Kalopita, Emmanouil Stamatakis, Dimitrios Valsamidis, George Daskalakis

**Affiliations:** 1 First Department of Obstetrics and Gynecology, Alexandra Hospital, National & Kapodistrian University of Athens, Athens, GRC; 2 Department of Anesthesiology and Pain Medicine, Alexandra Hospital, National & Kapodistrian University of Athens, Athens, GRC

**Keywords:** placenta accreta spectrum disorder, abnormally invasive placenta, postpartum hemorrhage, uterine atony, abnormal placentation, peripartum hysterectomy

## Abstract

Background

Although peripartum hysterectomy (PH) is a life-saving procedure in cases of abnormal placentation and postpartum hemorrhage, it can be associated with major obstetric and anesthetic complications. This retrospective study aimed to evaluate the incidence, etiology, perioperative anesthetic and obstetric management, complications, and fetal outcomes in women undergoing PH in a single tertiary referral hospital in Greece.

Methodology

This was a retrospective analysis of medical records of women who underwent emergency or elective PH in our hospital between January 2015 and December 2018.

Results

During the study period, 69 women who underwent a PH were identified. The incidence rate of elective and emergency PH was 4 and 1.2 per 1,000 deliveries, respectively. The main indication for PH was abnormal placentation (81.2%), followed by uterine atony (13%). Conversion to general anesthesia (GA) was performed in 21 (30.4%) cases.

Conclusions

This study showed a high prevalence of PH in our hospital compared to high-income countries. A neuraxial-only technique may be a safe alternative in individual cases of abnormal placentation. Conversion to GA can be reserved for complex surgical cases when massive hemorrhage is anticipated and, if possible, after the neonate has been delivered.

## Introduction

Peripartum hysterectomy (PH) is considered a life-saving procedure. Given that the uterus is removed and the subsequent high morbidity, this procedure is mostly reserved for cases of intractable obstetric hemorrhage not responsive to conservative surgical measures. In the past, uterine atony and uterine rupture were the main indications for emergency peripartum hysterectomy (EPH) [1]. However, this trend has changed over the past decades mainly due to the availability of blood transfusion and successful treatment of uterine atony with uterotonic agents, uterine massage, B-lynch sutures, ligation of the internal iliac artery, and uterine artery embolization. Since the 1980s, this trend has shifted, and abnormal placentation is now the main indication for PH. The reason for this is the higher rate of cesarean sections and the rise of in vitro fertilization/assisted reproductive techniques over the past decades [1,2].

Placenta accreta spectrum (PAS), previously known as morbidly adherent placenta, refers to the range of pathologic adherence of the placenta, including placenta increta, placenta percreta, and placenta accreta [3]. It is a potentially life-threatening condition during pregnancy associated with uncontrolled and intractable hemorrhage leading to hypovolemic shock, hysterectomy, multisystem organ failure, disseminated intravascular coagulation, or even maternal death. Given the increasing incidence and morbidity associated with PAS, anesthesiologists play a vital role in optimizing the maternal outcomes of these women. Recognized risk factors of PAS include previous cesarean sections, placenta previa, history of uterine curettage or myomectomy, and any procedure that could cause uterine scarring [4,5].

PH is performed in cases of uncontrolled obstetric hemorrhage where all conservative measures have failed. It results in permanent loss of fertility and is associated with high maternal morbidity due to concurrent intraoperative and postoperative complications. The implementation of massive transfusion protocols and the availability of interventional radiology techniques have significantly reduced the need for hysterectomy; however, the prevalence of the procedure remains high across different clinical settings [1,6]. Although the optimal management of PAS remains debatable, the International Federation of Gynecology and Obstetrics (FIGO) suggests that a prenatal diagnosis along with a multidisciplinary approach can improve maternal outcomes [7].

The objectives of this retrospective study were to evaluate the incidence and etiology of PH in a tertiary referral center for high-risk pregnancies in Athens, Greece. Moreover, the aim was to provide data regarding the perioperative anesthetic and obstetric strategies implemented in these cases and to compare the results of this study with relevant previously reported studies.

## Materials and methods

For this retrospective study, labor ward registries, operating theater records, and anesthetic charts of all parturients who underwent a PH over a four-year period (January 1, 2015, to December 31, 2018) were reviewed. Among the participants screened, we included only women who had undergone a PH after the 24th week of gestation and until six weeks after delivery. The study was performed in “Alexandra” General Hospital, which is a tertiary referral center for high-risk pregnancies. The study was approved by the Local Institutional Research Ethics Board (Scientific Ethical Committee) (Reg. No: 9th theme, 3rd Session, Approval Date: March 20, 2018).

Maternal age, gravidity, parity, gestational age, number of previous cesarean sections, indications for hysterectomy, perioperative anesthetic and obstetric management, pre- and postoperative hemoglobin and hematocrit levels, and neonatal Apgar scores in the first and fifth minute were recorded for each patient and newborn. Body mass index (BMI) was calculated as kg/m^2^. Additionally, intraoperative red blood cells (RBCs), fresh frozen plasma (FFP), platelets (PLT), tranexamic acid (TXA), human fibrinogen concentrate, prothrombin complex concentrate (PCC), recombinant activated factor VII (rFVIIa), crystalloid, and colloid transfusions were documented. Postoperative follow-up in the obstetric High Dependency Unit (HDU) along with admission to the Intensive Care Unit (ICU) was evaluated. Our obstetric HDU provides temporary maternal intensive care, including invasive monitoring with an arterial line but does not provide care for patients who are intubated or require a vasopressor infusion. The need for intraoperative consultation with a gynecologic oncologist or a urologist was also recorded. The type of placental invasion anomaly was retrieved using Doppler ultrasound or magnetic resonance imaging (MRI) reports and confirmed by histopathologic findings. In the majority of cases, the same team of three expert consultant obstetricians was involved.

Quantitative variables were expressed as mean (standard deviation, SD) or median (interquartile range, IQR). Qualitative variables are presented as absolute and relative frequencies. For the comparisons of proportions, the chi-square and Fisher’s exact tests were performed. Student’s t-tests were used for the comparison of mean values when the distribution was normal and the Mann-Whitney test for the comparison of median values when the distribution was non-normal. Paired Student’s t-test was performed for pre- and post-surgery comparison of hemoglobin and hematocrit levels. Data were analyzed using SPSS software for Windows version 22.0 (IBM Corp., Armonk, NY, USA). A p-value of <0.05 was considered statistically significant.

## Results

Over the four-year study period (2015-2018), 69 women underwent a PH in our hospital. During this period, a total of 16,672 women delivered, while 8,326 had a cesarean section, resulting in a cesarean section rate of almost 50%. The incidence rate of PH was 4 and 1.2 per 1,000 elective and emergency deliveries, respectively. The mean age of the parturients was 34.9 years (SD = 5.55), and the mean gestational age at the time of hysterectomy was 35 weeks (SD = 2.35). The mean BMI of women was 28.4 kg/m^2^ (SD = 5.1), and the median gravidity was 3 (IQR = 2-4). The majority of women (n = 61, 88.4%) had a previous cesarean section, of whom 27 (39.1%) had one previous cesarean and 34 (49.3%) had undergone two or more previous cesarean sections. Only eight (11.6%) women had an unscarred uterus. In our cohort, PH was planned preoperatively (elective PH) in 50 of 69 (72.5%) cases, while emergency hysterectomy was done in 19 out of 69 (27.5%) cases. The indications for PH were PAS (72.5%), uterine atony (13%), uterine rupture (1.4%), known malignancy (2.8%), and history of trachelectomy (1.4%) (Table 1).

**Table 1:** Maternal and clinical characteristics of cases. SD: standard deviation; IQR: interquartile range; BMI: body mass index; RBC: red blood cell; FFP: fresh frozen plasma; PLT: platelet; PCC: prothrombin complex concentrate; ICU: intensive care unit; HDU: high dependency unit

Age, mean (SD)		34.9 (5.6)
BMI, mean (SD)		28.4 (5.1)
Gestational age (weeks), mean (SD)		35 (2.4)
Parity, N (%)	1	3 (4.3)
	2	26 (37.7)
	3	20 (29.0)
	>3	20 (29.0)
Previous cesarean section, N (%)	0	8 (11.6)
	1	27 (39.1)
	2	19 (27.5)
	>2	15 (21.7)
Indications for peripartum hysterectomy, N (%)	Placenta accreta	7 (10.1)
	Placenta increta	31 (44.9)
	Placenta percreta	18 (26.1)
	Uterine atony	9 (13.0)
	Uterine rupture	1 (1.4)
	Ovarian cancer	1 (1.4)
	Uterine sarcoma	1 (1.4)
	History of trachelectomy (cervical cancer)	1 (1.4)
Intraoperative blood loss (mL), median (IQR)		3,771 (500–9,400)
Preoperative hemoglobin (g/dL), mean (SD)		9.3 (1.6)
Postoperative hemoglobin reduction (g/dL), mean (SD)		0.5 (1.8)
Intraoperative crystalloid fluids transfused, mean (SD)		3,792.3 (1,198.3)
Units of RBCs transfused, median (IQR)		4 (2–5)
Units of FFP transfused, median (IQR)		3 (2–4)
PLTs transfusion, N (%)		2 (2.9)
PCCs administration, N (%)		2 (2.9)
Tranexamic acid administration, N (%)		33 (47.8)
Fibrinogen administration, N (%)		14 (20.3)
Emergency cesarean section, N (%)		19 (27.5)
Gynecological oncologist in addition to obstetrician, N (%)		42 (60.9)
Urologist in addition to obstetrician, N (%)		6 (8.7)
Admission to the ICU, N (%)		4 (5.8)
Admission to the HDU, N (%)		66 (95.7)
Routine postpartum bed, N (%)		0 (0.0)
Duration of hospital stay (days), median (IQR)		10 (8–19)
Duration of postoperative hospital stay (days), median (IQR)		7 (6–9)

The most common maternal complication was febrile morbidity (15.9%). Wound infection and disseminated intravascular coagulation occurred in 7.2% and 4.3% of women, respectively. There was one case of intraoperative bladder injury and another of ureteral transection. Two patients required re-laparotomy, one for persistent intra-abdominal bleeding and another for adnexal torsion, both resulting in good resolution. Enterectomy with subsequent anastomosis was required in one patient. No maternal death was reported. Subtotal hysterectomy was performed in only two patients due to perioperative difficulties in excising the cervix.

Four patients (5.8%) required admission to the ICU, whereas the remaining were admitted to the HDU. More than half of the cases (60.9%) required intervention from a gynecologic oncologist due to advanced surgical complexity, while six required a urologist consultation. The mean duration of hospital stay after the procedure was 7.9 days (range = 3-32). The mean estimated blood loss during the procedure was 3,771 mL (range = 500-9,400 mL), and the mean preoperative hemoglobin level was 9.3 g/dL (SD = 1.6), with a mean reduction of 0.5 g/dL (SD = 1.8) after surgery (p = 0.029). All patients required a transfusion of at least one unit of RBCs, with the median number of RBCs transfused being four units (IQR = 2-5), while the median number of FFP transfused was three units (IQR = 2-4). TXA and fibrinogen were administrated in 47.8% and 20.3% of the parturients, respectively. Only two (2.9%) women received PLTs or PCCs. Recombinant factor VII was administered to none of the patients (Table 1).

Anesthetic data are displayed in Table 2. General anesthesia (GA) was induced according to the urgency of obstetric hysterectomy and expected blood loss. In 28/69 (40.6%) of the cases, neuraxial anesthesia was performed, with most of these patients (20/69, 29%) being treated with combined spinal-epidural (CSE) anesthesia. In the remaining 41 (59.4%) cases, GA was employed. Conversion to GA was performed in 21 out of 69 (30.4%) cases, mostly due to excessive hemorrhage. Anesthetic complications were reported in two (2.9%) cases and included bronchospasm and an allergic rash during transfusion. In 87% of the parturients, invasive blood pressure (BP) monitoring was established, while in 29%, a central venous catheter (CVC) was inserted. Seven postoperative analgesia regimens were identified, with the most common being a combination of epidural morphine and intravenous patient-controlled analgesia (PCA) (53.6%) (Table 2).

**Table 2:** Anesthetic data. CSE: combined spinal-epidural; GA: general anesthesia; RBC: red blood cell; NSAIDs: non-steroidal anti-inflammatory drugs; COX-2: cyclooxygenase-2; PCA: patient-controlled analgesia; IV: intravenous

Type of anesthesia		N (%)
Neuraxial		28 (40.6)
	CSE	20 (29.0)
	CSE + top-up epidural	1 (1.4)
	Double-space CSE	2 (2.9)
	Epidural	2 (2.9)
	Spinal	3 (4.3)
	Conversion to GA	21 (30.4)
GA		41 (59.4)
Induction to GA		N (%)
	Etomidate	1 (1.4)
	Propofol	16 (23.2)
	Thiopental	41 (59.4)
Intubation		N (%)
	Videolaryngoscope	1 (1.4)
	Direct endotracheal intubation	62 (89.9)
Maintenance of anesthesia		N (%)
	Desflurane	1 (1.4)
	Propofol	2 (2.9)
	Sevoflurane	59 (85.5)
Opioids		N (%)
	Fentanyl boluses	54 (78.3)
	Morphine-fentanyl boluses	7 (10.1)
Reported anesthetic complications		N (%)
	No complications	67 (97.1)
	Bronchospasm	1 (1.4)
	Allergic reaction after RBC transfusion	1 (1.4)
Remaining intraoperative anesthetic data		N (%)
	Extubated at the end of the case	60 (87.0)
	Vasoactive drugs	12 (17.4)
	Invasive blood pressure monitoring	60 (87.0)
	Internal jugular venous access	20 (29.0)
Postoperative analgesia		N (%)
	Epidural morphine ± ropivacaine 0.2%+ IV paracetamol + conventional NSAIDs/COX-2 inhibitorsv ± IV opioids	9 (13.0)
	Epidural morphine + IV PCA	37 (53.6)
	IV paracetamol + conventional NSAIDs/COX-2 inhibitors + IV PCA	9 (13.0)
	IV paracetamol + conventional NSAIDs/COX-2 inhibitors + IV strong opioids	13 (18.8)
	IV paracetamol + conventional NSAIDs/COX-2 inhibitors + IV weak opioids	5 (7.2)

Table 3 presents the association of RBC and FFP transfusions with clinical data. The number of RBC units transfused was significantly higher in patients who received GA, required invasive BP monitoring, were administered vasoactive drugs, or were admitted to the ICU. Furthermore, a significant positive association was found between the number of RBC units transfused, the volume of crystalloids administered, and the duration of postoperative hospital stay. The number of FFP units transfused was higher during an emergency cesarean section. Additionally, a greater amount of FFPs was administered to parturients who required vasopressors or a postoperative admission to the ICU (Table 3).

**Table 3:** Association of RBC, FFP, tranexamic acid, and fibrinogen administration with clinical data. RBC-FFP transfusions: +Kruskal-Wallis test; ++Mann-Whitney test; ‡Spearman’s correlation coefficient. Tranexamic acid-fibrinogen administration: +Student’s t-test; ++Mann-Whitney test; ‡Pearson’s chi-square test; ‡‡Fisher’s exact test. IQR: interquartile range; RBC: red blood cell; FFP: fresh frozen plasma; BMI: body mass index

		RBC transfusions		FFP transfusions		Tranexamic acid			Fibrinogen		
		Median (IQR)	P-value	Median (IQR)	P-value	No, N (%)	Yes, N (%)	P-value	No, N (%)	Yes, N (%)	P-value
Age, r^‡^		-0.17	0.154	-0.08	0.481	35 (5.5)	35.4 (5.7)	0.735^+^	35.2 (5.8)	34.8 (4.1)	0.786^+^
BMI, r^‡^		-0.11	0.441	-0.15	0.297	28.7 (5.2)	27.4 (4.7)	0.385^+^	27.8 (4.6)	29.7 (6.5)	0.273^+^
Parity	1-2	4 (2–5)	0.482^+^	3 (2–4)	0.978^+^	18 (56.3)	14 (43.8)	0.962^‡^	27 (84.4)	5 (15.6)	0.518^‡‡^
	3	4 (2–6)		3 (2–4)		11 (52.4)	10 (47.6)		15 (71.4)	6 (28.6)	
	>3	4 (3–5)		2 (2–4)		11 (55.0)	9 (45.0)		17 (85.0)	3 (15.0)	
Gestational age (weeks), r^‡^		-0.10	0.409	0.03	0.771	34.3 (3.4)	34 (4.8)	0.776^+^	34.3 (3.6)	33.5 (5.6)	0.530^+^
Previous cesarean section	0	2.5 (2–5)	0.423^+^	2 (2–4)	0.903^+^	5 (50.0)	5 (50.0)	0.865^‡^	7 (70.0)	3 (30.0)	0.680^‡‡^
	1	4 (2–5)		3 (2–4.5)		14 (50.0)	14 (50.0)		24 (85.7)	4 (14.3)	
	2	4 (2–6)		3 (2–4)		12 (60.0)	8 (40.0)		16 (80.0)	4 (20.0)	
	>2	4 (3–5)		2 (2–4)		9 (60.0)	6 (40.0)		12 (80.0)	3 (20.0)	
Emergency cesarean section	No	4 (2–5)	0.379^++^	2 (2–4)	0.007^++^	29 (58.0)	21 (42.0)	0.529^‡^	42 (84.0)	8 (16.0)	0.335^‡‡^
	Yes	4 (2–5)		4 (2–5)		11 (50.0)	11 (50.0)		16 (72.7)	6 (27.3)	
Duration of hospital stay (days), r^‡^		0.09	0.486	0.04	0.765	13.5 (8–22)	10 (-8–16)	0.318^++^	14.5 (9–21.5)	8 (7–13)	0.046^++^
Duration of postoperative hospital stay (days), r^‡^		0.26	0.034	0.17	0.173	7.5 (3.8)	8.1 (5.3)	0.618^+^	7.5 (3.5)	8.9 (7.3)	0.300^+^
Gynecological oncologist	No	3 (2–5)	0.057^++^	2 (2–4)	0.182^++^	16 (53.3)	14 (46.7)	0.834^‡^	24 (80.0)	6 (20.0)	0.882^‡^
	Yes	4 (3–6)		3 (2–4)		24 (55.8)	19 (44.2)		35 (81.4)	8 (18.6)	
Urologist	No	4 (2–5)	0.207^++^	2 (2–4)	0.083^++^	37 (55.2)	30 (44.8)	1.000^‡‡^	55 (82.1)	12 (17.9)	0.324^‡‡^
	Yes	4.5 (3–6)		4.5 (3–6)		3 (50.0)	3 (50.0)		4 (66.7)	2 (33.3)	
Type of anesthesia	Neuraxial	3 (2–4)	0.027^++^	2.5 (2–4)	0.385^++^	16 (57.1)	12 (42.9)	0.750^‡^	25 (89.3)	3 (10.7)	0.147^‡^
	General	4 (3–5)		3 (2–4)		24 (53.3)	21 (46.7)		34 (75.6)	11 (24.4)	
Conversion to general anesthesia	No	4 (2–5)	0.444^++^	2 (2–4)	0.511^++^	31 (59.6)	21 (40.4)	0.193^‡^	40 (76.9)	12 (23.1)	0.324^‡‡^
	Yes	4 (2–4)		3 (2–4)		9 (42.9)	12 (57.1)		19 (90.5)	2 (9.5)	
Admission to ICU	No	4 (2–5)	0.007^++^	2 (2–4)	0.006^++^	38 (56.7)	29 (43.3)	0.321^‡‡^	57 (85.1)	10 (14.9)	0.018^‡‡^
	Yes	6 (5.5–7.5)		6 (4.5–7.5)		1 (25.0)	3 (75.0)		1 (25.0)	3 (75.0)	
Vasoactive drugs	No	4 (2–5)	0.044^++^	2.5 (2–4)	0.049++	35 (58.3)	25 (41.7)	0.113^‡^	51 (85.0)	9 (15.0)	0.048^‡‡^
	Yes	5 (3.5–6.5)		5 (2–6)		4 (33.3)	8 (66.7)		7 (58.3)	5 (41.7)	
Invasive blood pressure monitoring	No	2 (2–3)	0.002^++^	2 (1–3)	0.073^++^	5 (50.0)	5 (50.0)	0.741^‡‡^	8 (80.0)	2 (20.0)	1.000^‡‡^
	Yes	4 (2.5–5)		3 (2–4)		34 (56.7)	26 (43.3)		49 (81.7)	11 (18.3)	
Intravenous fluids administered	Colloids, r^‡^	0.20	0.092	0.20	0.096	0 (0–500)	500 (0–500)	0.230^++^	0 (0–00)	500 (0–1,000)	0.030^++^
	Crystalloids, r^‡^	0.30	0.011	0.14	0.261	3,769.2 (1,101.2)	3,820.3 (1,324.6)	0.860^+^	3,669.3 (1,177.9)	4,292.9 (1,191.3)	0.081^+^
APGAR score	First minute, r‡	-0.08	0.506	0.13	0.292	7.5 (1.7)	6.9 (2.5)	0.301^+^	7.4 (1.8)	6.7 (3.1)	0.311^+^
	Fifth minute, r‡	-0.01	0.947	0.21	0.170	8.1 (1.9)	7.2 (3.1)	0.247^+^	8 (2.1)	6.7 (3.8)	0.170^+^

TXA was administered to 33 (47.8%) patients; however, there was no correlation with any of the data presented in Table 3. Fibrinogen administration was recorded in 14 (20.3%) patients. Parturients admitted to the ICU or administered vasoactive drugs intraoperatively received fibrinogen in most cases. Additionally, there was a positive correlation between the amount of colloids administered and the replacement of fibrinogen.

## Discussion

In our hospital, the reported incidence of PH and EPH is 4 and 1.2 per 1,000 deliveries, respectively. This is considerably higher than the rates reported in other studies, which ranged from 0.2 to 0.45 per 1,000 deliveries [1,8,9]. This is likely attributed to two factors. First, the number of cesarean sections performed in Greece is significantly higher in relation to the population compared with other countries [10]. The second reason is the fact that our hospital is a referral center for PAS cases.

Although uterine atony has traditionally been described as the main risk factor for PH, there is a change in trends over the past decades, indicating placental pathology as the leading cause [8]. It has been well established that CS is the main cause of abnormal placentation leading to PAS and eventually hysterectomy [2]. The risk of PAS is higher among women with an obstetrical history of previous cesarean sections, and it fluctuates from 0.3% for one previous cesarean section to as high as 6.74% for women with five or more previous cesarean sections [11]. Placenta previa further increases this risk to 3%, 11%, 40%, 61%, and 67% for the first, second, third, fourth, and fifth cesarean deliveries, respectively [4,12]. In our hospital, the rate of cesarean sections was 49.9% during the study period, which is significantly higher compared to previously reported studies [1,13]. Placenta previa was diagnosed in 36 of our patients. In total, PAS was confirmed in 56 patients (32 placenta previa), of whom 54 had one or more previous cesarean sections in their obstetric history. It is evident that, in our population, the main risk factor for PAS and PH is the number of previous cesarean sections.

The mean birth weight of the neonates was 2,395 g (SD = 516.2), which is significantly lower than the mean birth weight of a term pregnancy. The main reason for this difference is prematurity. Women with placenta previa and accreta are at a considerably higher risk for preterm labor due to obstetric complications, such as vaginal bleeding and massive hemorrhage. Moreover, to prevent any adverse outcomes associated with placental pathology, it has been advised that pregnant women deliver before the 36th week of gestation. Indeed, the American College of Obstetricians and Gynecologists (ACOG) suggested that cesarean sections and consequent PH should be performed between the 34th and 36th week of pregnancy to ensure maternal and fetal wellbeing [14]. First- and fifth-minute Apgar scores were 7.44 (SD = 1.68) and 8.07 (SD = 1.87), respectively. Two stillbirths were recorded in our study population. Compared to the general population, these values are low; however, they are justified if we take into consideration the mean gestational week at delivery in our population [15,16].

The arterial blood pressure is impaired by regional anesthesia which provokes sympathetic blockade. Despite the fact that the same can also be caused by GA, due to its better titrability compared to regional anesthesia, in cases of anticipated major intraoperative blood loss, GA may be employed. One should keep in mind the option of conversion to GA, especially in the presence of significant hemorrhage, airway management difficulties, and severe acidosis. In our study, conversion to GA was performed in 21 (30%) cases, which is in accordance with the findings of a retrospective study by Lilker et al., where 29% of the patients with PAS underwent conversion to GA due to excessive bleeding [17]. Riveros-Peres et al. suggested that, after the umbilical cord has been clamped, conversion of CSE to GA should be used as a routine anesthetic practice, especially when a protocol of massive hemorrhage is being employed [18]. The application of a neuraxial-only technique can avert certain complications of GA, provide better pain control, and minimize the need for transfusion [19]. In our study, only 40.6% of patients initially received a neuraxial-only technique. However, Markley et al. recently reported that 84% of the parturients who had undergone cesarean delivery for placenta previa had successfully received neuraxial anesthesia, implying that the neuraxial-only technique can be employed in all similar cases [20]. It must be highlighted that placenta previa cases undergoing cesarean sections, such as those mentioned in the aforementioned study, and PH cases are two different situations, and extrapolation of conclusions based on these findings may not be appropriate. However, these data, although not conclusive, are suggestive of the role of neuraxial anesthesia in such cases. Additionally, Hong et al. compared general versus epidural anesthesia for cesarean sections in the presence of placenta previa and concluded that the latter is superior with regard to maternal hemodynamic status and blood loss [21]. It is clear that the ideal anesthetic technique in cases of PAS remains controversial and possible conversion from a neuraxial technique to GA should be individualized. Because management of neuraxial-induced sympathectomy in cases of obstetric hemorrhage is challenging, anesthesiologists should be prepared to convert to GA in cases of anticipated surgical complexity or airway difficulties [20].

When comparing our results with those of a similar study also conducted in our hospital, no differences were noted regarding the estimated blood loss and transfusion strategies [22]. All of our patients received a blood transfusion, whereas 73% required more than two units of blood products. Additionally, more than half of the patients met the criteria of a massive transfusion protocol. This is in accordance with the findings of other studies, which showed that PH is associated with an increased need for massive fluid resuscitation strategies [23]. In our study, the number of RBC units transfused was greater in women who received GA, required invasive BP monitoring, and those who received vasoactive drugs or were admitted to the ICU. Invasive monitoring is a prerequisite for the effective control of massive hemorrhage, and an arterial line was placed in the majority of our patients before the start of the procedure [24,25].

Regarding the perioperative administration of TXA, no association was found with the parameters presented in Table 3. According to the WOMAN trial, early administration of TXA is associated with a reduction in mortality, without having any maternal adverse effects. These findings suggest that the administration of TXA should be initiated as soon as heavy bleeding is detected [26,27]. In our study, such correlation was not demonstrated, which may be attributed to the fact that no deaths were recorded, as well as to the fact that our population size was small. Moreover, fibrinogen should be administered in cases of massive hemorrhage to maintain a level of ≥2 g/dL [28]. In our study, fibrinogen administration was associated with a higher rate of admission to the ICU and increased administration of vasoactive drugs and colloids, but with a shorter duration of hospital stay. This may indicate that, even though fibrinogen is employed in cases of massive hemorrhage, it can be beneficial for the faster recovery of patients. Cases with anticipated major bleeding may require massive transfusion resuscitation protocols and could benefit from early fibrinogen replacement to prevent postpartum hemorrhage-induced coagulopathy [29].

This study has certain limitations. First, the sample size was not large enough to develop a multidisciplinary algorithm protocol. Moreover, our institution does not support the routine use of cell salvage in cases of abnormally invasive placenta. Furthermore, none of our cases had access to an interventional radiology suite for arterial embolization. However, we truly believe that our study findings will facilitate the adaptation of best practices regarding anesthetic and obstetric interventions, especially in low-resource clinical settings. Regarding the conversion of CSE to GA, we advocate that this could be reserved for complex surgical cases where massive hemorrhage is anticipated, preferably after the delivery of the neonate.

## Conclusions

In this study, previous cesarean section and placenta previa were associated with PAS, the main indication for PH, a procedure associated with significant maternal morbidity and prolonged hospital stay. Efforts should be made to reduce the number of unnecessary cesarean deliveries. Guideline adaptation along with a highly experienced maternity center with a coordinated care team can result in better maternal and fetal outcomes. A neuraxial-only technique may be a safe alternative in individual cases of abnormal placentation.
